# Supplementary material for: P-TRAP: a Panicle Trait Phenotyping tool
Source: BMC Plant Biol. 2013 Aug 29;13:122. doi: 10.1186/1471-2229-13-122 (PMC3848748; doi:10.1186/1471-2229-13-122)
Supplement: Additional file 7 — P-TRAP source code. The Netbeans project that contains the source code of the application. [file 1471-2229-13-122-S7.zip › javadoc.zip!/javadoc/org-bioalg-imageprocessor/org/bioalg/imageprocessor/AImage.html]

AImage (Image Processor)


JavaScript is disabled on your browser.


- Package
- Class
- Tree
- Deprecated
- Index
- Help

*org.bioalg.imageprocessor 1.0*

- Prev Class
- Next Class

- Frames
- No Frames

- All Classes

- Summary:
- Nested |
- Field |
- Constr |
- Method

- Detail:
- Field |
- Constr |
- Method


org.bioalg.imageprocessor

## Class AImage

- java.lang.Object
- - org.bioalg.imageprocessor.AImage

- ---

    

  ```
  public class AImage
  extends java.lang.Object
  ```

  The main class used for image processing of the project. Please in case of
  any use of this code cite the corresponding authors of the article(s) where
  this code is published with.
  This handles the binary and grayscale images.

  Author:
  :   aanjos and Faroq Al-tam

- - ### Nested Class Summary

    Nested Classes

    | Modifier and Type | Class and Description |
    | --- | --- |
    | `static class` | `AImage.CONNECTIVITY` |
  - ### Constructor Summary

    Constructors

    Constructor and Description

    `AImage(AImage otherAI)`

    Copy constructor

    `AImage(double[][] mx,
    int bitDepth)`

    From a double matrix with a given bitDepth

    `AImage(int[][] mx,
    int bitDepth)`

    From int matrix with a given bitdepth

    `AImage(int nRows,
    int nCols,
    int bitDepth)`
  - ### Method Summary

    Methods

    | Modifier and Type | Method and Description |
    | --- | --- |
    | `AImage` | `AND(AImage img)` The logical ANDing process between this image and the input image. |
    | `double` | `bilinearInterp(double u, double v)` The bilinear interpolation of two values |
    | `static boolean` | `convertType(java.io.File sourceFile, java.io.File destinationFile, java.lang.String imageType)` Convert between image types. |
    | `void` | `convolve2d(double[][] kernel)` The 2D convolution of the current image. |
    | `void` | `denormalize()` Restores the normalized image from [0-1] to [0-maxInt] |
    | `AImage` | `fastMeanThresholding(int kernerlsize, double cval)` An optimized version of the mean local threshold method |
    | `boolean` | `fillholes(int serachkernel)` A fill-holes algorithm based on finding a suitable seed on the background, fill the background by a color then replace this color in the filled image by backgorund and replace the rest by foreground color! |
    | `AImage` | `fillsmallholes(int serachkernel, int smallesthole)` Small holes filling algorithm, it is based on some mathematical basic operations and the holes filling classical approach |
    | `java.util.ArrayList<java.util.ArrayList<java.awt.Point>>` | `findconnectedcompnents(AImage aImage, int minimumsize)` Find the connected components in the image. |
    | `int` | `floodfill(java.awt.Point initial, java.awt.Point end, int bkcolor, int fillcolor)` Implements the breadth-first flood fill algorithm |
    | `int` | `floodfill2(java.awt.Point initial, java.awt.Point end, int bkcolor, int fillcolor)` Implements the depth-first flood fill algorithm |
    | `void` | `gaussianBlur(double sigma)` The Gaussian blurring. |
    | `int[][]` | `getcircularNxNmatrix(int i, int j, int radius)` Return a circular matrix centered at row i and column j of the original image. |
    | `int[][]` | `getcircularNxNmatrixROI(int i, int j, java.util.ArrayList<java.awt.Point> RIO, int radius)` Return a circular matrix centered at row i and column j of the original image. |
    | `java.util.ArrayList<java.awt.Point>` | `getconnectedObject(java.awt.Point initial, java.awt.Point end, int bkcolor, int fillcolor)` Implements the depth-first flood fill algorithm to find a connected object |
    | `java.util.ArrayList<java.awt.Point>` | `getconnectedObject2(java.awt.Point initial, java.awt.Point end, int bkcolor, int fillcolor)` |
    | `int` | `getDepth()` |
    | `double` | `getEntropy()` Calculate the entropy of the image's histogram. |
    | `java.util.ArrayList<java.awt.Point>` | `getFGPixels(java.util.ArrayList<java.awt.Point> ROI)` Return the white pixels in a ROI. |
    | `int[]` | `getfirstvector(int i, int j, int n)` Get the first vector of a kernel of nXn kernel |
    | `double[]` | `getHistogram()` Calculate the histogram of the image. |
    | `int[]` | `getHistogramInt()` Return an integer histogram of the image. |
    | `int[]` | `getlastvector(int i, int j, int n)` Return the last vector of a kernel of nXn matrix |
    | `double[][]` | `getMatrix()` Return the image matrix. |
    | `int[][]` | `getNbyNsubmatrix(int i, int j, int n)` Return an nXn matrix of the image given a pixel position (i,j) |
    | `AImage` | `getNormMaxMin()` Returns a normalized image, with intensities scaled to max-min intensities. |
    | `AImage` | `getNormMaxMin(int maxbound)` Get a normalized version of the image scaled from 0 to a bound. |
    | `double` | `getRC(int r, int c, double border)` Get the pixel value at a given row and column. |
    | `AImage` | `getScaledAImage(double scaleFactor)` Returns a new object of AImage scaled by scaleFactor |
    | `AImage` | `getSubImage(int startX, int startY, int endX, int endY)` Return a sub image given four points. |
    | `double` | `getsumOfROI(java.util.ArrayList<java.awt.Point> ROI)` Get the sum of the pixels in a ROI region |
    | `double` | `getTotalIntensities()` The possible number of intensities according to the bitdepth |
    | `int` | `height()` The height of the image |
    | `void` | `invert()` |
    | `boolean` | `isclean(int[][] mat)` Return true if the matrix contains all zeros |
    | `boolean` | `isdarkBackGround()` Given a binary image indicate whether it has a dark or white background. |
    | `double` | `max()` |
    | `AImage` | `meanLowpass(int kernerlsize)` Fast mean smoothing method. |
    | `void` | `meanLowpassing(int ksize, double value)` A blurring mean filter |
    | `AImage` | `meanThresholding(int kernerlsize, double cval)` **Deprecated.** |
    | `double` | `myLog(double base, double val)` |
    | `void` | `normalize()` Normalize the image with respect to the possible number if intensities. |
    | `void` | `normalizeHistogram(double[] hist)` Normalize the histogram and put it between 0 and 1. |
    | `AImage` | `OR(AImage img)` The logical ORing process. |
    | `void` | `printMatrix()` |
    | `void` | `putMark(java.awt.Point initial, int size, int fillcolor)` Put a given rectangular mark with a given color |
    | `void` | `removecolor(int colorvlaue, int fillcolor)` Replace the pixels of a given color by another color. |
    | `void` | `removeComponent(java.util.ArrayList<java.awt.Point> component)` Remove a given connected component. |
    | `void` | `sauvolaThresholding(int kernerlsize, double kvalue)` The Sauvola's local thresholding |
    | `void` | `setbyThreshold(int i, int j, double threshold)` Threshold a given pixel by a threshold |
    | `void` | `setDepth(int depth)` |
    | `void` | `setRC(int r, int c, double val, java.lang.String from)` Set the pixel's value at row r and column to another value val. |
    | `void` | `smoothMedianNxN(int radius)` The median filter. |
    | `AImage` | `subtract(AImage img)` Subtract and image form this image |
    | `void` | `toBinary(double threshold)` Simple global threshold segmentation method |
    | `int` | `validatepixels(java.util.ArrayList<java.awt.Point> points)` To calcuate how many pixels in the "points" param correspond to white pixels. |
    | `int` | `validatePixels(int[][] kernel)` The same as { |
    | `int` | `width()` The width of the image |
    | `void` | `zeroOneImage()` Convert a binary image to a zero-one image. |

    - ### Methods inherited from class java.lang.Object

      `clone, equals, finalize, getClass, hashCode, notify, notifyAll, toString, wait, wait, wait`

- - ### Constructor Detail


    - #### AImage

      ```
      public AImage(int nRows,
            int nCols,
            int bitDepth)
      ```


    - #### AImage

      ```
      public AImage(AImage otherAI)
      ```

      Copy constructor


    - #### AImage

      ```
      public AImage(int[][] mx,
            int bitDepth)
      ```

      From int matrix with a given bitdepth

      Parameters:
      :   `mx` -
      :   `bitDepth` -


    - #### AImage

      ```
      public AImage(double[][] mx,
            int bitDepth)
      ```

      From a double matrix with a given bitDepth

      Parameters:
      :   `mx` -
      :   `bitDepth` -
  - ### Method Detail


    - #### getTotalIntensities

      ```
      public double getTotalIntensities()
      ```

      The possible number of intensities according to the bitdepth

      Returns:
      :   a double value represents the possible intensities.


    - #### getMatrix

      ```
      public double[][] getMatrix()
      ```

      Return the image matrix.

      Returns:
      :   a 2D double matrix


    - #### height

      ```
      public int height()
      ```

      The height of the image

      Returns:
      :   integer value.


    - #### width

      ```
      public int width()
      ```

      The width of the image

      Returns:
      :   integer value


    - #### getNormMaxMin

      ```
      public AImage getNormMaxMin()
      ```

      Returns a normalized image, with intensities scaled to max-min
      intensities.

      Returns:
      :   A new `AImage` object.


    - #### getScaledAImage

      ```
      public AImage getScaledAImage(double scaleFactor)
      ```

      Returns a new object of AImage scaled by scaleFactor

      Parameters:
      :   `scaleFactor` - double value that determines the scaling factor.

      Returns:
      :   A new object of `AImage`


    - #### bilinearInterp

      ```
      public double bilinearInterp(double u,
                          double v)
      ```

      The bilinear interpolation of two values

      Parameters:
      :   `u` - double, the first value
      :   `v` - double, the second value.

      Returns:
      :   double value.


    - #### printMatrix

      ```
      public void printMatrix()
      ```


    - #### getRC

      ```
      public double getRC(int r,
                 int c,
                 double border)
      ```

      Get the pixel value at a given row and column.

      Parameters:
      :   `r` - the row
      :   `c` - the column
      :   `border` - if =-1 and r or c are out of the image domain, the function
          returns the border value.

      Returns:
      :   a double value of the intensity at the given pixel.


    - #### setRC

      ```
      public void setRC(int r,
               int c,
               double val,
               java.lang.String from)
      ```

      Set the pixel's value at row r and column to another value val.

      Parameters:
      :   `r` - the row
      :   `c` - the column
      :   `val` - the value to set at r and c.
      :   `from` - where this function is called from.


    - #### convolve2d

      ```
      public void convolve2d(double[][] kernel)
      ```

      The 2D convolution of the current image.

      Parameters:
      :   `kernel` - a 2D double matrix representing the convolution kernel.


    - #### normalize

      ```
      public void normalize()
      ```

      Normalize the image with respect to the possible number if intensities.


    - #### denormalize

      ```
      public void denormalize()
      ```

      Restores the normalized image from [0-1] to [0-maxInt]


    - #### zeroOneImage

      ```
      public void zeroOneImage()
      ```

      Convert a binary image to a zero-one image. The one is the 255 in this
      case.


    - #### smoothMedianNxN

      ```
      public void smoothMedianNxN(int radius)
      ```

      The median filter.

      Parameters:
      :   `radius` - the radius of the median kernel.


    - #### setDepth

      ```
      public void setDepth(int depth)
      ```


    - #### getDepth

      ```
      public int getDepth()
      ```


    - #### getHistogram

      ```
      public double[] getHistogram()
      ```

      Calculate the histogram of the image.

      Returns:
      :   a double array of the image intensities pdf distribution.


    - #### getHistogramInt

      ```
      public int[] getHistogramInt()
      ```

      Return an integer histogram of the image.

      Returns:
      :   an integer array.


    - #### normalizeHistogram

      ```
      public void normalizeHistogram(double[] hist)
      ```

      Normalize the histogram and put it between 0 and 1.

      Parameters:
      :   `hist` -


    - #### myLog

      ```
      public double myLog(double base,
                 double val)
      ```


    - #### getEntropy

      ```
      public double getEntropy()
      ```

      Calculate the entropy of the image's histogram.

      Returns:
      :   a double value.


    - #### getSubImage

      ```
      public AImage getSubImage(int startX,
                       int startY,
                       int endX,
                       int endY)
      ```

      Return a sub image given four points.

      Parameters:
      :   `startX` - the start row
      :   `startY` - the start column
      :   `endX` - the end row
      :   `endY` - the end column

      Returns:
      :   a new image


    - #### getNbyNsubmatrix

      ```
      public int[][] getNbyNsubmatrix(int i,
                             int j,
                             int n)
      ```

      Return an nXn matrix of the image given a pixel position (i,j)

      Parameters:
      :   `i` - the row
      :   `j` - the colu,m
      :   `n` - the width of the summatrix

      Returns:
      :   an nXn integer submatrix


    - #### getcircularNxNmatrixROI

      ```
      public int[][] getcircularNxNmatrixROI(int i,
                                    int j,
                                    java.util.ArrayList<java.awt.Point> RIO,
                                    int radius)
      ```

      Return a circular matrix centered at row i and column j of the original
      image.

      Parameters:
      :   `i` - the row
      :   `j` - the column
      :   `RIO` - an `ArrayList` of points that represents the Region Of
          Interest.
      :   `radius` - the radius of the matrix

      Returns:
      :   a 2D integer matrix.


    - #### getcircularNxNmatrix

      ```
      public int[][] getcircularNxNmatrix(int i,
                                 int j,
                                 int radius)
      ```

      Return a circular matrix centered at row i and column j of the original
      image.

      Parameters:
      :   `i` - the row
      :   `j` - the column
      :   `radius` - the radius of the matrix

      Returns:
      :   a 2D integer matrix.

      See Also:
      :   `getcircularNxNmatrixROI(int, int, java.util.ArrayList, int)`


    - #### getfirstvector

      ```
      public int[] getfirstvector(int i,
                         int j,
                         int n)
      ```

      Get the first vector of a kernel of nXn kernel

      Parameters:
      :   `i` - the row
      :   `j` - the column
      :   `n` - the width of the kernel

      Returns:
      :   an integer array of the retrieved pixels


    - #### getlastvector

      ```
      public int[] getlastvector(int i,
                        int j,
                        int n)
      ```

      Return the last vector of a kernel of nXn matrix

      Parameters:
      :   `i` - the row
      :   `j` - the column
      :   `n` - the width of the matrix

      Returns:
      :   a vector of size n

      See Also:
      :   `getfirstvector(int, int, int)`


    - #### getsumOfROI

      ```
      public double getsumOfROI(java.util.ArrayList<java.awt.Point> ROI)
      ```

      Get the sum of the pixels in a ROI region

      Parameters:
      :   `ROI` - an `ArrayList` that represents the RIO points.

      Returns:
      :   a double value.


    - #### toBinary

      ```
      public void toBinary(double threshold)
      ```

      Simple global threshold segmentation method

      Parameters:
      :   `threshold` - the user defined threshold


    - #### meanLowpassing

      ```
      public void meanLowpassing(int ksize,
                        double value)
      ```

      A blurring mean filter

      Parameters:
      :   `ksize` - the width of the kernel
      :   `value` - the value to use in convolution.


    - #### max

      ```
      public double max()
      ```


    - #### sauvolaThresholding

      ```
      public void sauvolaThresholding(int kernerlsize,
                             double kvalue)
      ```

      The Sauvola's local thresholding

      Parameters:
      :   `kernerlsize` - the size of the w\*w neighborhood
      :   `kvalue` - the k parameter of the method


    - #### removecolor

      ```
      public void removecolor(int colorvlaue,
                     int fillcolor)
      ```

      Replace the pixels of a given color by another color.

      Parameters:
      :   `colorvlaue` - color to replace
      :   `fillcolor` - color to add


    - #### setbyThreshold

      ```
      public void setbyThreshold(int i,
                        int j,
                        double threshold)
      ```

      Threshold a given pixel by a threshold

      Parameters:
      :   `i` - the row
      :   `j` - the column
      :   `threshold` - the intensity threshold


    - #### meanThresholding

      ```
      public AImage meanThresholding(int kernerlsize,
                            double cval)
      ```

      Deprecated.

      The mean local thresholding.

      Parameters:
      :   `kernerlsize` - the size of the w\*w neighborhood
      :   `cval` - the c parameter of the method


    - #### fastMeanThresholding

      ```
      public AImage fastMeanThresholding(int kernerlsize,
                                double cval)
      ```

      An optimized version of the mean local threshold method

      Parameters:
      :   `kernerlsize` -
      :   `cval` -

      Returns:
      :   a segmented image

      See Also:
      :   `meanThresholding`


    - #### floodfill

      ```
      public int floodfill(java.awt.Point initial,
                  java.awt.Point end,
                  int bkcolor,
                  int fillcolor)
      ```

      Implements the breadth-first flood fill algorithm

      Parameters:
      :   `initial` - the ground-zero for the flooding
      :   `end` - if needed is the last point the flood will stop at, default
          null
      :   `fillcolor` - the filling value.


    - #### floodfill2

      ```
      public int floodfill2(java.awt.Point initial,
                   java.awt.Point end,
                   int bkcolor,
                   int fillcolor)
      ```

      Implements the depth-first flood fill algorithm

      Parameters:
      :   `initial` - the ground-zero of the flooding
      :   `end` - if needed is the last point the flood will stop at, default is
          null
      :   `fillcolor` - the filling value.


    - #### putMark

      ```
      public void putMark(java.awt.Point initial,
                 int size,
                 int fillcolor)
      ```

      Put a given rectangular mark with a given color

      Parameters:
      :   `initial` - the center of the mark
      :   `size` - the width of the mark
      :   `fillcolor` - the value to fill the mark with.


    - #### getconnectedObject

      ```
      public java.util.ArrayList<java.awt.Point> getconnectedObject(java.awt.Point initial,
                                                           java.awt.Point end,
                                                           int bkcolor,
                                                           int fillcolor)
      ```

      Implements the depth-first flood fill algorithm to find a connected
      object

      Parameters:
      :   `initial` - the ground-zero of the flooding
      :   `end` - if needed is the last point the flood will stop at, default is
          null
      :   `fillcolor` - the filling value.


    - #### getconnectedObject2

      ```
      public java.util.ArrayList<java.awt.Point> getconnectedObject2(java.awt.Point initial,
                                                            java.awt.Point end,
                                                            int bkcolor,
                                                            int fillcolor)
      ```


    - #### validatepixels

      ```
      public int validatepixels(java.util.ArrayList<java.awt.Point> points)
      ```

      To calcuate how many pixels in the "points" param correspond to white
      pixels. Used to calculate the exact area of a given object after cleaning
      some undesirable parts

      Parameters:
      :   `points` -

      Returns:
      :   the number of white pixels in the point set.


    - #### validatePixels

      ```
      public int validatePixels(int[][] kernel)
      ```

      The same as {

      Parameters:
      :   `kernel` - the pixels

      Returns:
      :   the number of white pixels

      See Also:
      :   `} but by using 2D matrix
          instead of a point list.`


    - #### subtract

      ```
      public AImage subtract(AImage img)
      ```

      Subtract and image form this image

      Parameters:
      :   `img` - the image to subtract.

      Returns:
      :   a new image of the subtraction result.


    - #### AND

      ```
      public AImage AND(AImage img)
      ```

      The logical ANDing process between this image and the input image.

      Parameters:
      :   `img` - the input image

      Returns:
      :   a new image as a result of the ANDing task.


    - #### OR

      ```
      public AImage OR(AImage img)
      ```

      The logical ORing process.

      Parameters:
      :   `img` - the input image.

      Returns:
      :   a new image.


    - #### isclean

      ```
      public boolean isclean(int[][] mat)
      ```

      Return true if the matrix contains all zeros

      Parameters:
      :   `mat` - a 2D matrix

      Returns:
      :   true if the matrix is all zero, false otherwise.


    - #### findconnectedcompnents

      ```
      public java.util.ArrayList<java.util.ArrayList<java.awt.Point>> findconnectedcompnents(AImage aImage,
                                                                                    int minimumsize)
      ```

      Find the connected components in the image.

      Parameters:
      :   `aImage` - the input image.
      :   `minimumsize` - the minimum size of the component to accept.

      Returns:
      :   the list of the found components


    - #### fillsmallholes

      ```
      public AImage fillsmallholes(int serachkernel,
                          int smallesthole)
      ```

      Small holes filling algorithm, it is based on some mathematical basic
      operations and the holes filling classical approach

      Parameters:
      :   `serachkernel` - The width of the search kernel
      :   `smallesthole` - A threshold to fill only small holes

      Returns:


    - #### removeComponent

      ```
      public void removeComponent(java.util.ArrayList<java.awt.Point> component)
      ```

      Remove a given connected component. Its pixels will all turn to black.

      Parameters:
      :   `component` - the input component to remove.


    - #### fillholes

      ```
      public boolean fillholes(int serachkernel)
      ```

      A fill-holes algorithm based on finding a suitable seed on the
      background, fill the background by a color then replace this color in the
      filled image by backgorund and replace the rest by foreground color!

      Parameters:
      :   `serachkernel` - a search kernel for a starting seed for filling the
          background


    - #### invert

      ```
      public void invert()
      ```


    - #### meanLowpass

      ```
      public AImage meanLowpass(int kernerlsize)
      ```

      Fast mean smoothing method. A very fast and optimized version of mean low
      passing.

      Parameters:
      :   `kernerlsize` -

      Returns:
      :   a smoothed version of the input image


    - #### convertType

      ```
      public static boolean convertType(java.io.File sourceFile,
                        java.io.File destinationFile,
                        java.lang.String imageType)
      ```

      Convert between image types.

      Parameters:
      :   `sourceFile` - the source image
      :   `destinationFile` - the destination image
      :   `imageType` - the type to convert to.

      Returns:
      :   ture if success, false otherwise.


    - #### getNormMaxMin

      ```
      public AImage getNormMaxMin(int maxbound)
      ```

      Get a normalized version of the image scaled from 0 to a bound.

      Parameters:
      :   `maxbound` - the bound to scale to.

      Returns:
      :   a normalized version of the input image.


    - #### gaussianBlur

      ```
      public void gaussianBlur(double sigma)
      ```

      The Gaussian blurring.

      Parameters:
      :   `sigma` - the Gaussian sigma.


    - #### isdarkBackGround

      ```
      public boolean isdarkBackGround()
      ```

      Given a binary image indicate whether it has a dark or white background.

      Returns:


    - #### getFGPixels

      ```
      public java.util.ArrayList<java.awt.Point> getFGPixels(java.util.ArrayList<java.awt.Point> ROI)
      ```

      Return the white pixels in a ROI.

      Parameters:
      :   `ROI` - the region of interest

      Returns:
      :   A list of points where the pixels are white.


- Package
- Class
- Tree
- Deprecated
- Index
- Help

*org.bioalg.imageprocessor 1.0*

- Prev Class
- Next Class

- Frames
- No Frames

- All Classes

- Summary:
- Nested |
- Field |
- Constr |
- Method

- Detail:
- Field |
- Constr |
- Method
